# Complete mitochondrial genome of the Longsheng Feng chicken (*Gallus gallus*)

**DOI:** 10.1080/23802359.2020.1791753

**Published:** 2020-07-20

**Authors:** Jingjing Gu, Sheng Li

**Affiliations:** a College of Animal Science and Technology, Hunan Agricultural University, Changsha, China; b Hunan Key Laboratory for Genetic Improvement of Animals, Changsha, China; c Hunan Engineering Research Center of Poultry Production Safety, Changsha, China; d Maxun Biotechnology Institute, Changsha, China

**Keywords:** Longsheng Feng chicken, mitochondrial genome, next-generation sequencing

## Abstract

In this study, the complete mitochondrial genome of Longsheng Feng chicken (*Gallus gallus*) was obtained by using a next-generation sequencing method for the first time. The complete mitochondrial genome sequence of Longsheng Feng chicken contains 2 ribosomal RNAs, 13 protein-coding genes, 22 transfer RNA genes and one control region. This work provides valuable genetic data for chicken mitochondrial researches and phylogenetic studies of domestic chickens.

Longsheng Feng chicken is an indigenous chicken breed with a long term breeding history by Yao people in South China. It has been placed on the national protection list of livestock and poultry in 2009. Longsheng Feng chickens have compact bodies, feathered legs and a variety of feather color patterns. In this study, Longsheng Feng chicken sample was collected from its original breeding center – Longsheng County (25.79 N and 110.01 E), Guangxi province, China. The Longsheng Feng chicken specimen (Voucher No. LS150224) was stored at −80 °C in the Museum of Hunan provincial key laboratory for genetic improvement of domestic animal, Changsha, China. The total genomic DNA was extracted from this muscle specimen and used as input material to make sequencing libraries. The prepared libraries then sequenced on the Illumina Hiseq 2500 sequencing machine. We generated 10.94 Gb raw sequencing data in total and submitted those reads to the NCBI Sequence Read Archive (SRA) with accession number SRR4302062. The assembled complete mitochondrial genome of Longsheng Feng chicken has been deposited in Genbank with accession number MT635914.

We annotated the complete mitogenome sequence of Longsheng Feng chicken using tRNAscan-SE 2.0 (Chan and Lowe 2019) and MITOS (Bernt et al. 2013). The total length of the mitogenome is 16,784 bp and the overall nucleotide composition is 30.2% A, 23.7% T, 32.5% C and 13.5% G with biased toward A + T (53.9%). The mitochondrial genome of Longsheng Feng chicken shows a typical vertebrate double strands circular structure and contains 2 ribosomal RNA genes (rRNAs), 13 protein-coding genes (PCGs), 22 transfer RNA genes (tRNAs), and 1 noncoding control region (D-loop region). Most PCGs (12 out of 13) use ATGs as the initiation codons while only *COX1* uses GTG. These PCGs have four types of termination codons, which are TAA, TAG, AGG, and an incomplete termination codon “T–”, which is the 5′ terminal of adjacent gene (Anderson et al. 1981). The 22 tRNA genes were distributed among rRNA and PCGs.

Phylogenetic relationship of Longsheng Feng chicken is inferred from the neighbor-joining (NJ) phylogenetic tree with other chicken breeds by using Mega 7.0 (Kumar et al. 2016). The NJ tree is constructed using 37 complete mitochondrial genome sequences of diverse chicken breeds with 1000 bootstrap replicates. The NJ tree (Figure 1) shows the Longsheng Feng chicken is close related with Xuefeng, Guangxi Partridge, Lverwu and Nandan. However, Jiangbian has the greatest genetic distance with Longsheng Feng chicken. This work provides valuable genetic data for chicken mitochondrial researches and phylogenetic studies of domestic chickens.

**Figure 1.**
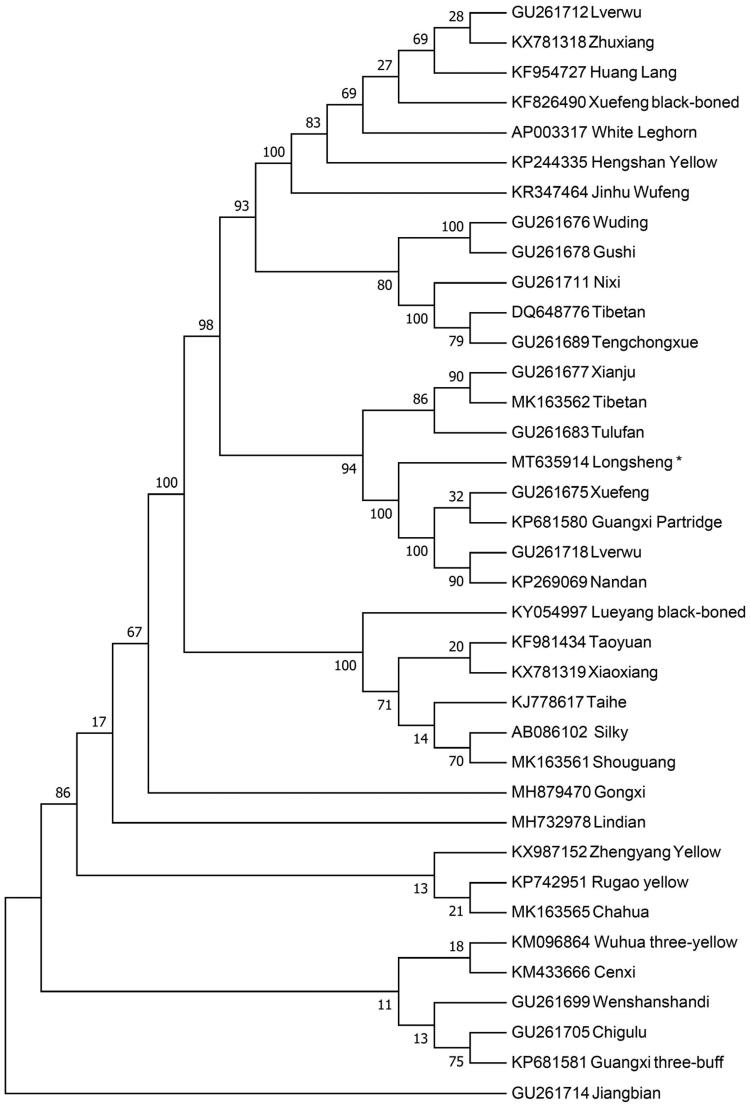
The neighbor-joining tree based on the complete mitochondrial DNA sequence of 37 chicken breeds. GenBank accession numbers are given before the species name.

## Data Availability

The sequence data that support the findings of this study are openly available in the NCBI Sequence Read Archive (SRA) at http://www.ncbi.nlm.nih.gov/sra/ with accession number SRR4302062. The complete mitochondrial genome of Longsheng Feng chicken (Gallus gallus) is openly available in GenBank at http://www.ncbi.nlm.nih.gov/genbank with accession number MT635914.

## References

[CIT0001] Anderson S, Bankier AT, Barrell BG, de Bruijn MH, Coulson AR, Drouin J, Eperon IC, Nierlich DP, Roe BA, Sanger F, et al. 1981. Sequence and organization of the human mitochondrial genome. Nature. 290(5806):457–464. 7219534 10.1038/290457a0

[CIT0002] Bernt M, Donath A, Juhling F, Externbrink F, Florentz C, Fr itzsch G, Putz J, Middendorf M, Stadler PF. 2013. MITOS: improved de novo metazoan mitochondrial genome annotation. Mol Phylogenet Evol. 69(2):313–319. 22982435 10.1016/j.ympev.2012.08.023

[CIT0003] Chan P, Lowe T. 2019. tRNAscan-SE: searching for tRNA genes in genomic sequences. Methods Mol Biol. 1962:1–14. 31020551 10.1007/978-1-4939-9173-0_1 PMC6768409

[CIT0004] Kumar S, Stecher G, Tamura K. 2016. MEGA7: molecular evolutionary genetics analysis version 7.0 for bigger datasets. Mol Biol Evol. 33(7):1870–1874. 27004904 10.1093/molbev/msw054 PMC8210823

